# Facile Arsenazo III-Based Assay for Monitoring Rare Earth Element Depletion from Cultivation Media for Methanotrophic and Methylotrophic Bacteria

**DOI:** 10.1128/AEM.02887-17

**Published:** 2018-04-02

**Authors:** Carmen Hogendoorn, Paula Roszczenko-Jasińska, N. Cecilia Martinez-Gomez, Johann de Graaff, Patrick Grassl, Arjan Pol, Huub J. M. Op den Camp, Lena J. Daumann

**Affiliations:** a Department of Microbiology, Radboud University, Nijmegen, the Netherlands; b Department of Microbiology and Molecular Genetics, Michigan State University, East Lansing, Michigan, USA; c Department of Chemistry, Ludwig-Maximilians-Universität München, Munich, Germany; Goethe University Frankfurt am Main

**Keywords:** Arsenazo III, Methylacidiphilum fumariolicum, Methylobacterium extorquens, cerium, europium, lanthanides, lanthanum, methanotrophy, methylotrophy, rare earth elements

## Abstract

Recently, methanotrophic and methylotrophic bacteria were found to utilize rare earth elements (REEs). To monitor the REE content in culture media of these bacteria, we have developed a rapid screening method using the Arsenazo III (AS III) dye for spectrophotometric REE detection in the low μM (0.1 to 10 μM) range. We designed this assay to follow La^III^ and Eu^III^ depletion from the culture medium by the acidophilic verrucomicrobial methanotroph Methylacidiphilum fumariolicum strain SolV. The assay can also be modified to screen the uptake of other REEs, such as Pr^III^, or to monitor the depletion of La^III^ from growth media in neutrophilic methylotrophs such as Methylobacterium extorquens strain AM1. The AS III assay presents a convenient and fast detection method for REE levels in culture media and is a sensitive alternative to inductively coupled plasma mass spectrometry (ICP-MS) or atomic absorption spectroscopy (AAS).

**IMPORTANCE** REE-dependent bacterial metabolism is a quickly emerging field, and while the importance of REEs for both methanotrophic and methylotrophic bacteria is now firmly established, many important questions, such as how these insoluble elements are taken up into cells, are still unanswered. Here, an Arsenazo III dye-based assay has been developed for fast, specific, and sensitive determination of REE content in different culture media. This assay presents a useful tool for optimizing cultivation protocols, as well as for routine REE monitoring during bacterial growth without the need for specialized analytical instrumentation. Furthermore, this assay has the potential to promote the discovery of other REE-dependent microorganisms and can help to elucidate the mechanisms for acquisition of REEs by methanotrophic and methylotrophic bacteria.

## INTRODUCTION

**FIG 1:**
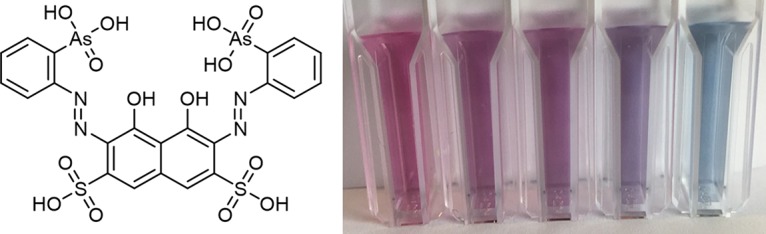
The Arsenazo III dye and the colored response to increasing lanthanum(III) concentrations at pH 2.8 (from left to right, 0 to 10 μM lanthanum(III)).

Rare earth elements (REEs) are relevant for Gram-negative methylotrophic bacteria, which utilize these strong Lewis acids in the active sites of alcohol dehydrogenases, including methanol dehydrogenases (MDHs), such as XoxF, and ethanol dehydrogenases, such as ExaF (1). The biological relevance of REEs has been demonstrated in MDHs isolated from Methylobacterium radiotolerans, Methylobacterium extorquens, and Bradyrhizobium sp. strain MAFF211645, which are dependent on La^III^ and Ce^III^ (2–4). In 2014, Pol et al. published the first, and only reported to date, crystal structure of an XoxF MDH, isolated from the verrucomicrobial methanotroph Methylacidiphilum fumariolicum SolV (5). This microorganism strictly depends on the presence of REEs for its growth, and this discovery marked the start of a rapid development in the field of study of REE-dependent methanotrophs and methylotrophs (5–18). Additionally, REEs also have a catalytic role for the ethanol dehydrogenase ExaF from *Mb. extorquens* AM1, the most efficient ethanol dehydrogenase reported to date (1). The accommodation of an REE seems to require the presence of a specific aspartate residue near the catalytic site (6). REE biochemistry is not unique to methylotrophs, as nonmethylotrophic organisms, such as Pseudomonas putida strain KT2440, can also express REE-dependent alcohol dehydrogenases, such as PedH (19). Due to their low solubility, it was long deemed impossible for REEs to be of biological relevance. And while the field of study of REE-dependent microorganisms is now quickly emerging, many important questions are still open. How do microorganisms manage to mobilize REEs, and how are these elements taken up into cells (8)? An REE-sensing mechanism has been proposed in bacteria that are able to express both MxaF (a calcium-dependent MDH) and XoxF MDH (3, 16). However, little is known about the uptake systems of these bacteria. Whether there might be similar uptake mechanisms as in Fe^III^-transporting siderophores (20) or REE-specific ion channels still needs to be investigated. REEs are important components for the development of digital and renewable energy technologies, but current recycling and separation methods are not efficient, and they are expensive. New microbiological approaches for the recovery and remediation of the technologically indispensable REEs have been demonstrated, where REEs, such as lanthanum or dysprosium, were bioaccumulated by Citrobacter and Penidiella strains (21, 22). Further, Bonificio and Clarke reported a method for the separation of the heavy REEs thulium, ytterbium, and lutetium using cells of Roseobacter (23). While it was not investigated whether the REEs were only adsorbed or absorbed by the cells, depletion and subsequent release of the three heaviest REEs into the medium were demonstrated (23). The methods employed to monitor REE depletion from media or to determine REE content in biological and environmental samples usually involve inductively coupled plasma optical emission spectroscopy (ICP-OES), inductively coupled plasma mass spectrometry (ICP-MS), or atomic absorption spectroscopy (AAS), with the last method being the least sensitive, with high limits of detection (24). Sample preparation for these methods can be time-consuming, and analytical facilities might not be readily available. While some REEs, such as europium(III) and terbium(III), display inherent luminescent properties that can be exploited for quantification, the equipment needed and the optimization of the assays in aqueous solution can be cumbersome due to the need for specific antenna ligands and unwanted luminescence quenching by water molecules (25). Chromogenic compounds for the determination of REEs have been around for more than 50 years and provide a promising alternative to these methods (26, 27). Of these compounds, the metallochromic indicator Arsenazo III (AS III; Fig. 1) has been shown to detect concentrations of REEs as low as 0.01 μg/ml (22, 27–29). This dye forms different colored complexes with a number of divalent and trivalent ions under acidic pH conditions (Fig. 1). REEs readily hydrolyze at an acidic pH and can thus be analyzed in the presence of calcium and other metal cations (30). While no crystal structure of an AS III complex with metal ions is available, it has been proposed that AS III forms 1:1 complexes with metals and that only one arseno group is involved in metal binding (28, 29). In order to monitor depletion of REEs from culture media of acidophilic and neutrophilic bacteria we have developed an assay that allows for fast detection of these metal ions at a concentration ranging from an REE content of 0.1 to 10 μM, a range that is often used for cultivation. Here, we present an assay based on AS III to screen for La^III^ depletion during the cultivation of the acidophilic methanotroph M. fumariolicum SolV. This assay can easily be modified to determine concentrations of other REEs, such as europium or praseodymium. It is also suited to analyze culture media of bacteria that grow at a neutral pH, such as *Mb. extorquens* AM1. However, in that case, it is necessary to adjust the pH of the medium prior to the measurements. Chrome azurol S was also evaluated. However, the pH optimum for sensitive detection of REEs with this dye is between pH 7.0 and 7.5, where other ions, such as iron(III) and copper(II), will significantly interfere with the assay. In addition, the sensitivity of this dye is generally lower (31, 32).

## RESULTS AND DISCUSSION

### pH dependence of the AS III assay.

**FIG 2:**
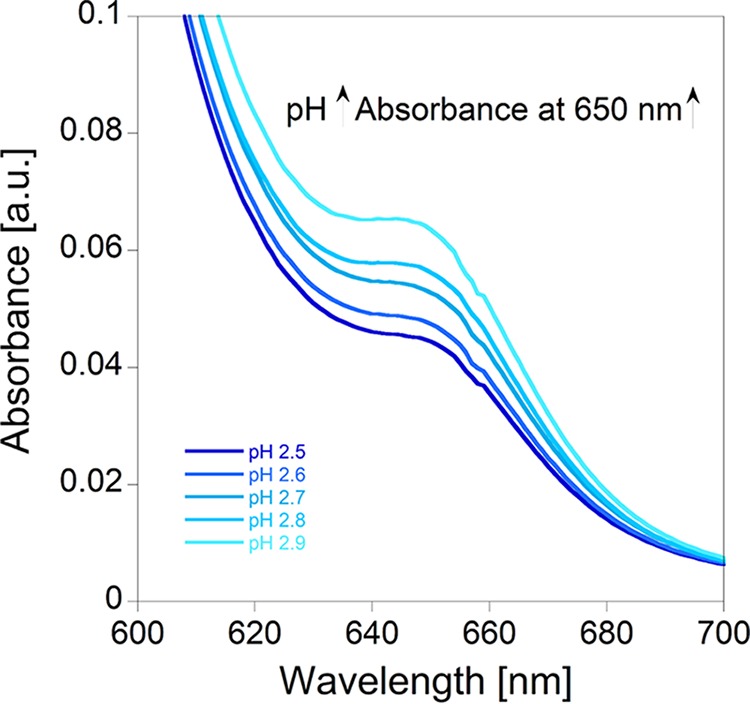
pH-dependence of the AS III assay. The solutions contained 1 μM La^III^, 15 μM AS III, 490 μl M. fumariolicum SolV growth medium, and 500 μl citrate/phosphate buffer. The buffer solutions were adjusted to pH 2.5, 2.6, 2.7, 2.8, and 2.9.

Both the AS III dye and its response to metal ions are pH sensitive. The AS III-La complex has a pH optimum for spectrophotometric analysis, shown in Fig. 2. While the AS III-La^III^ absorbance at 650 nm will increase with increasing pH, as demonstrated in Fig. 2, at higher pH other metal ions will also bind to AS III and interfere with the assay. Hence, to avoid interference of ions such as calcium(II) or copper(II), the REE content should be determined at a pH below 2.9. A pH between 2.7 and 2.8 was found to be an excellent compromise between sensitivity and selectivity. Free AS III exhibits one broad peak between 450 and 700 nm, at 535 nm. The AS III-La^III^ complex exhibits a maximum absorbance at 650 nm. For concentrations higher than 2 μM, an additional peak can be detected at 600 nm under certain conditions. This observation has also been made by Fernandez-Gavarron et al., who detected an additional peak at 597 nm when using a mixture of 25 μM AS III and 10 μM La at pH 3.1 (30). Since the pH of the cultivation medium varies during microbial growth, pH must be monitored and adjusted for the assay. This can be achieved by using a citrate/phosphate buffer system (pH 2.8). A variation in pH of more than 0.5 point within the measurement series will lead to an inaccurate photometric readout. In addition to the higher specificity for REEs, an acidic pH also ensures the enhanced solubility of REE ions in the presence of phosphate ions. The solubility of the REE phosphates is low, and at neutral pH, REE phosphates will precipitate readily from solution, especially in the absence of chelators like citrate or nitrilotriacetic acid (33).

### Metal ion dependence of the AS III assay.

**FIG 3:**
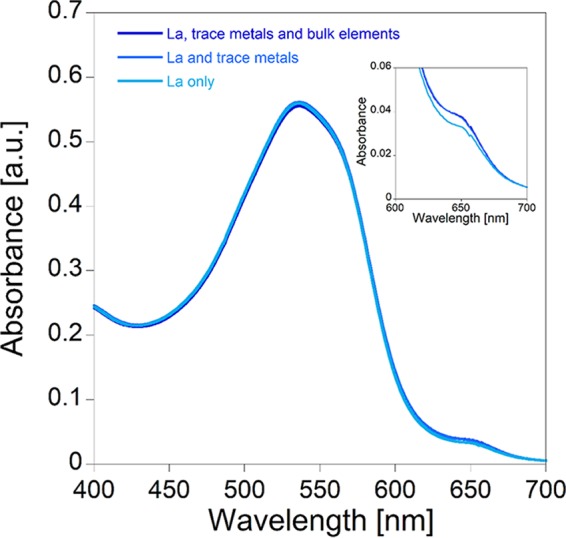
Dependence of AS III-La^III^-absorption at constant AS III (15 μM) and La^III^ (1 μM) concentrations and pH 2.8, with different “background” metals. In addition to 500 μl buffer (citrate/phosphate, pH 2.8), the solutions contained 490 μl M. fumariolicum SolV growth medium (trace and bulk elements), 490 μl M. fumariolicum SolV trace element solution, or 490 μl Millipore water.

**FIG 4:**
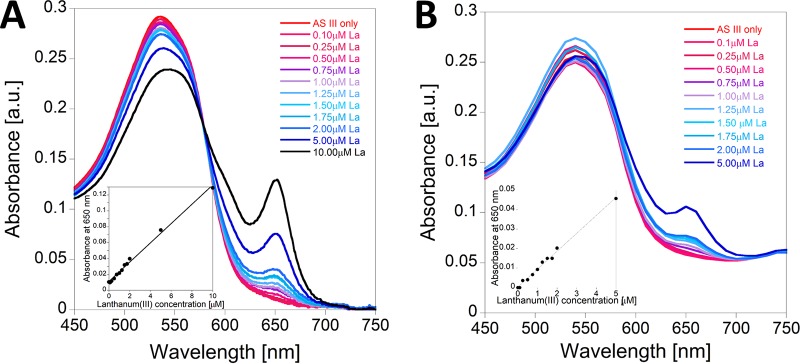
(A) La^III^ titration to a solution of 10 μM AS III in a mixture of citrate/phosphate buffer (pH 2.8) and M. fumariolicum SolV growth medium stock solution. (B) La^III^ titration to a solution of 10 μM AS III in a mixture of citrate/phosphate buffer (pH 2.8) and MP growth medium stock solution. Insets show plots of absorbance at 650 nm against increasing lanthanum(III) concentrations.

**FIG 5:**
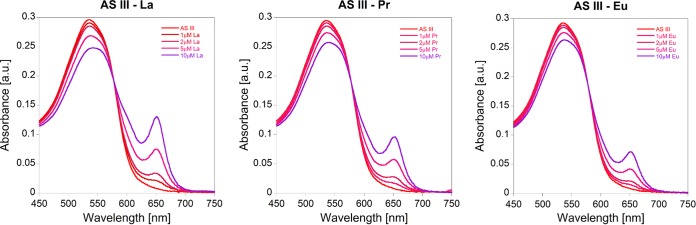
La^III^, Pr^III^, and Eu^III^ titrations to solutions of 10 μM AS III in a mixture of citrate/phosphate buffer (pH 2.8) and M. fumariolicum SolV growth medium stock solution.

We investigated whether the depletion of the bulk and trace elements from the medium during bacterial growth can impact the AS III-La^III^ absorbance readout at 650 nm. Figure 3 demonstrates that the elements of the bulk medium solution of M. fumariolicum SolV have only a very small impact on AS III-La^III^ absorption. To increase accuracy, it is thus recommended to perform a calibration curve with the growth medium stock solution. A step-by-step description of the calibration curve protocol can be found in Table 1. AS III-La^III^ absorbance follows a linear correlation from 0 to 2 μM. At higher concentrations of La^III^ (up to 10 μM) this correlation is strongly dependent on the conditions of the assay (e.g., pH, presence of buffer, and presence of other metal ions). Figure 4A shows the spectrophotometric titration of 0.1 to 10 μM La^III^ to a solution of 10 μM AS III in citrate/phosphate buffer (pH 2.8) in the presence of bulk medium and trace elements of M. fumariolicum SolV. In Fig. 4B, the titration of La^III^ to a solution of 10 μM AS III in citrate/phosphate buffer (pH 2.8) in the presence of minimal PIPES [piperazine-*N*,*N*′-bis(2-ethanesulfonic acid)] (MP) medium, as used for the cultivation of methylotrophs, is shown. Different REE ions can be screened using this assay. However, individual calibration curves have to be recorded for each derivative, as the intensity of the absorption at 650 nm varies within the series (Fig. 5).

**TABLE 1:** General protocol for the AS III assay

Stage	Step no.	Description^*a*^
Stock solution prepn	1	Prepare a 1 mM Arsenazo III stock solution in Millipore water.
	2	Prepare citric acid/phosphate buffer. For 100 ml, combine 84.15 ml of a 0.1 M citric acid solution and 15.85 ml of a 0.2 M Na_2_HPO_4_ solution. The final pH of this buffer solution should be 2.8.
	3	Prepare growth medium stock solutions. The pH of the growth medium solution of strain M. fumariolicum SolV is adjusted to 2.7–2.8 with sulfuric acid if neccesary. For neutrophilic methylotrophs like *Mb. extorquens* AM1, the MP medium was used. The pH of this growth medium solution is 6.7 and thus needs to be adjusted to pH 2.7–2.8 with 5 M hydrochloric acid. After acidifying the medium, centrifugation is recommended to remove precipitated PIPES buffer.
	4	Prepare a 1 mM La^III^ stock solution by dissolving LaCl_3_·7H_2_O in Millipore water. Note that plastic, as well as glass, containers can either take up or release lanthanides into the medium, so this solution should be prepared fresh for the calibration curve measurements (27).
Calibration sample prepn	5	Prepare dilutions for the calibration curve (for example, 0, 0.1, 0.25, 0.5, 0.75, 1, 1.5, and 2 μM La^III^ in growth medium stock solutions). Check the pH of these calibration solutions; it should be between pH 2.7–2.8.
Calibration curve	6	Transfer 500 μl of the citrate/phosphate buffer (pH 2.8) to a cuvette and add 490 μl of medium solution (0 μM La) or the respective calibration (0.1–2 μM La) solution. Record a blank on the UV-Vis instrument on this solution.
	7	Add 10 μl of the 1 mM Arsenazo III solution, mix, and record the UV-Vis spectrum. Note the absorbance at 650 nm.
	8	Add another 20 μl of Arsenazo III stock. Record UV-Vis spectrum. Note absorbance at 650 nm.
	9	Repeat steps 6 to 8 for the entire dilution series. Plot absorbance vs La^III^ concn.^*b*^
Sample measurement	10	Transfer 500 μl of the citrate/phosphate buffer (pH 2.8) into a cuvette and add 490 μl of the growth medium solution to be tested. Ensure that the pH is around 2.7–2.8, adjusting the pH of the sample prior to this step if necessary. Record a blank on the UV-Vis instrument on this solution.
	11	Add 10 μl of the 1 mM Arsenazo III solution and record the UV-Vis spectrum. Note absorbance at 650 nm.
	12	Add another 20 μl of Arsenazo III stock. Record UV-Vis spectrum. Note absorbance at 650 nm.
	13	Determine La^III^ concn from calibration curves.

a MP medium, minimal PIPES [piperazine-*N*,*N*′-bis(2-ethanesulfonic acid)] medium; UV-Vis, UV-visible.

b For La^III^ concentrations higher than 2 μM, the absorbance readout of the 30 μM AS III has been found to be more reliable. When working with lower concentrations than 2 μM, it is advisable to use the calibration curve obtained for 10 μM AS III.

### Assay evaluation to monitor La^III^ and Eu^III^ depletion during M. fumariolicum SolV cultivation.

**FIG 6:**
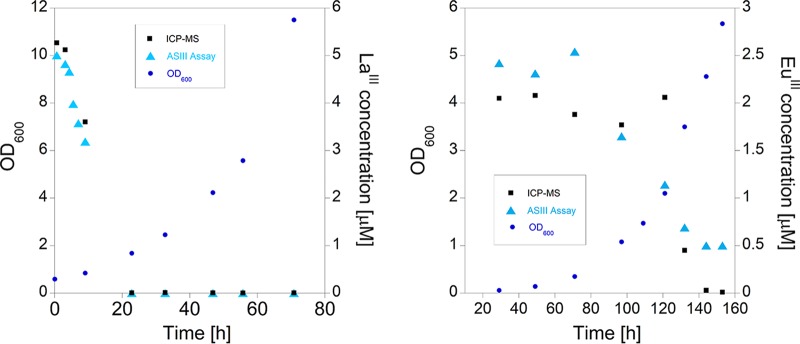
Optical density of M. fumariolicum SolV culture measured at different time points of the cultivation, as well as La^III^ (initial concentration at beginning of cultivation, 5 μM) and Eu^III^ (initial concentration at beginning of cultivation, 2 μM) content, as determined by ICP-MS and with the AS III assay (30 μM). The different timelines that were used here are a result of the fact that the growth rate of M. fumariolicum SolV with La^III^ is known to be faster than that with Eu^III^ (5).

To evaluate the AS III assay during microbial growth, depletion of La^III^ from the medium was followed during cultivation of M. fumariolicum SolV using the protocol described in Table 1. The initial La^III^ concentration was 5 μM, and the decrease in absorbance (650 nm), which reflected a depletion of the AS III-La complex, was observed at different time points during microbial growth (Fig. 6). In addition, the increase in biomass was monitored by optical density measurements (optical density at 600 nm [OD_600_]) and the La^III^ content of the medium was also analyzed by ICP-MS (Fig. 6). Furthermore, the cultivation with the slightly smaller REE Eu^III^ was carried out under these conditions. The results demonstrate that this assay can be used for the detection of a variety of REEs during microbial growth and that it provides a reliable alternative to ICP-MS. It is interesting to note that in addition to different growth rates with La^III^ and Eu^III^, the depletions of these metal ions by M. fumariolicum SolV follow different timelines, an observation that we are currently investigating.

### Assay evaluation to monitor La^III^ depletion during *Mb. extorquens* AM1 cultivation.

**FIG 7:**
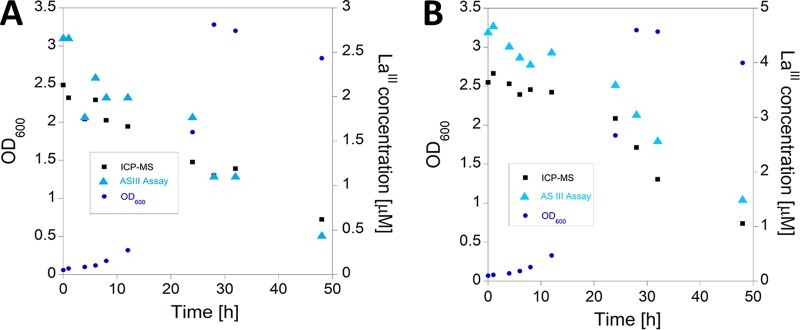
Optical density of *Mb. extorquens* AM1 culture measured at different time points of the cultivation and La^III^ contents as determined by AS III assay and ICP-MS. (A) Initial La concentration at beginning of cultivation, 2 μM. (B) Initial La concentration at beginning of cultivation, 4 μM.

In order to evaluate the precision of the AS III assay in monitoring La^III^ depletion by *Mb. extorquens* strain AM1, a mutant strain in which the *mxaF* gene is disrupted was used. This ensured that growth was entirely dependent on the presence of lanthanum in the culture medium (14). Depletion of 2 and 4 μM La^III^ from MP medium (34) during bacterial growth was monitored using the protocol described in Table 1. REE consumption was determined by AS III assay at different time points during *Mb. extorquens* AM1 growth and compared with the results obtained from the same samples by ICP-MS. The growth was monitored by optical density measurements (OD_600_). Figure 7 demonstrates that the AS III assay can also be used to monitor depletion of La^III^ by *Mb. extorquens* AM1.

### Conclusion.

Overall the ICP-MS and AS III measurements show a good correlation. It is interesting to note that the uptake mechanisms of the two bacterial species investigated here seem to be different. M. fumariolicum SolV depletes most REEs before growth starts (especially with earlier REEs, such as La), while *Mb. extorquens* AM1 shows a linear decrease in REE content over the whole growth period. We present here a quick and simple assay for direct determination of REE concentrations in the growth media of methanotrophic and methylotrophic bacteria using the Arsenazo III azo dye. The assay can be used for different REEs, although it was observed that the larger the REE, the more sensitive the assay. REE depletion can be monitored for both acidophilic and neutrophilic bacterial strains. Control of the pH in the calibration curve samples and test solutions is essential for the accuracy of the REE content determination.

## MATERIALS AND METHODS

Arsenazo III (AS III) and the REE salts were purchased from Sigma-Aldrich and used in the experiments without further purification. Initially, the dye (85% purity) was further purified using a published protocol (35). However, no significant change in REE detection was observed, and thus the dye was used as received. It is recommended to store the AS III solution in a dark vial and prepare a fresh solution every week. The overall absorbance of this dye solution will slightly decrease over time; hence, the calibration curve measurements and the assay should preferably be conducted on the same day with the same AS III stock solution. LaCl_3_·7H_2_O was of 99.999% purity. EuCl_3_·6H_2_O and PrCl_3_·6H_2_O were obtained in 99.99% purity. Buffer, metal stock solutions, and dye stock solutions were made up in deionized Millipore water. Additional treatment of the Millipore water with Chelex was not necessary. The assay was tested in Hellma quartz Suprasil cuvettes, as well as with disposable cuvettes (polymethyl methacrylate [PMMA]) and 96-well plates, and all were found to be suitable for this assay. The assay was tested on conventional diode array and xenon flash lamp UV-visible (UV-Vis) spectrometers, which can both be used, since AS III does not show significant decomposition during the short duration time of the assay when subjected to UV light. The assay can also be conducted using a microplate reader (e.g., BioTek EpochII; Winooski, VT).

### Growth medium for the acidophilic verrucomicrobial methanotroph M. fumariolicum SolV.

In the case of strain M. fumariolicum SolV, the final element solution contained the bulk elements (bulk medium solution) 0.2 mM MgCl_2_·6H_2_O, 1 mM Na_2_SO_4_, 2 mM K_2_SO_4_, 4 mM (NH_4_)_2_SO_4_, 1 mM NaH_2_PO_4_·H_2_O, and 0.2 mM CaCl_2_·2H_2_O and the trace elements 2 μM FeSO_4_·7H_2_O, 0.1 μM ZnSO_4_·7H_2_O, 0.1 μM CoCl_2_·6H_2_O, 2 μM MnCl_2_·4H_2_O, 3 μM CuSO_4_·5H_2_O, 0.1 μM NiCl_2_·6H_2_O, and 0.1 μM Na_2_MoO_4_·2H_2_O. The stock solution of the trace elements was dissolved in 1 to 2% sulfuric acid (vol/vol) before addition to the bulk medium solution. Usually, 10× and 1,000× concentrated solutions of bulk and trace elements were prepared and diluted together as needed.

### Cultivation of M. fumariolicum SolV.

 Batch cultivation with 2 μM EuCl_3_ was performed in a 10-liter fermenter (Applikon, Schiedam, Netherlands). The liquid volume was 5 liters and the temperature was set to 55°C. The pH was kept at 2.8 using 0.2 M NaOH. A total of 8 to 100 ml/min methane was supplied to the reactor. The air supply was 50 to 1,200 ml/min, and the agitation speed was between 400 and 1,000 rpm, in order to maintain a dissolved oxygen concentration of up to 10% air saturation. Samples were taken approximately every 12 h and were centrifuged at 2,770 × *g* for 20 min at room temperature. The clear supernatant was transferred to clean 15-ml centrifuge tubes stored at 4°C prior to analysis. One part of the supernatant was transferred to a clean 1.5-ml Eppendorf tube and used for the Arsenazo dye III assay. The remaining supernatant was used for ICP-MS analysis. All analyses were performed at the end of the batch experiments. Batch cultivation with 5 μM LaCl_3_ was performed in a 500-ml fermenter with a working volume of 400 ml. The bioreactor was operated at 55°C with a stirring rate of 700 rpm, using a stirrer bar. The pH was kept at 2.8 using 0.2 M NaOH. Ten milliliters per minute of gas containing 10% CH_4_ and 3% CO_2_, and 8% O_2_ was supplied to the reactor. Samples for the Arsenazo III dye assay were taken approximately every 2 h for the first 8 h of the experiment, and for the remaining time period, samples were taken every 12 h. Samples for the Arsenazo III dye assay were centrifuged at 20,238 × *g* for 10 min at room temperature. The supernatant was transferred to a clean Eppendorf tube and stored at −20°C prior to analysis. Samples for ICP-MS were taken at the start, after 8 h, and then approximately every 12 h and were centrifuged at 2,770 × *g* for 20 min at room temperature, and the clear supernatant was stored at 4°C prior to analysis. All analyses were performed at the end of the batch experiments. To analyze europium or lanthanum content in the SolV culture medium with ICP-MS, 10 ml of the clear supernatant was collected and passed through by 0.2-μm filter and acidified with 65% nitric acid to a final concentration of 1%. After sample preparation, metal analysis was performed using an inductively coupled plasma mass spectrometer (ICP-MS) (X series; Thermo Scientific) at the Faculty of Science, Radboud University (Nijmegen).

### Minimal PIPES (MP) growth medium for neutrophilic methylotrophs like *Mb. extorquens* AM1.

Minimal PIPES growth medium consisted of 30 mM PIPES, 1.45 mM K_2_HPO_4_·3H_2_O, 1.88 mM NaH_2_PO_4_·H_2_O, 0.5 mM MgCl_2_·6H_2_O, 8 mM (NH_4_)_2_SO_4_, 45.6 μM Na_3_C_6_H_5_O_7_·2H_2_O, 1.2 μM ZnSO_4_·7H_2_O, 1 μM MnCl_2_·4 H_2_O, 18 μM FeSO_4_·7H_2_O, 2 μM (NH_4_)_6_Mo_7_O_24_·4H_2_O, 1 μM CuSO_4_·5H_2_O, 2 μM CoCl_2_·6H_2_O, 0.33 μM Na_2_WO_4_·2H_2_O, 20 μM CaCl_2_, and 125 mM methanol (34).

### Cultivation of *Mb. extorquens* AM1.

One-hundred-milliliter *Mb. extorquens* AM1 cultures (*mxaF* mutant strain) (14) were grown in MP medium (34) containing 125 mM methanol as a carbon source and 2 μM or 4 μM LaCl_3_ in a flask at 30°C on a rotary shaker at 150 rpm for 48 h. Samples (5 ml each) were collected at time points of 0, 1, 4, 6, 8, 12, 24, 28, 32, and 48 h and centrifuged at 4,696 × *g* for 10 min at 4°C. Supernatants were filtered using a 0.2-μm syringe filter and acidified with 5 M HCl. Precipitated PIPES buffer was removed by centrifuging for 15 min at 4,696 × *g* at room temperature. Collected supernatants were then used either for the Arsenazo III assay or for ICP-MS analysis. For ICP-MS analysis, samples were diluted 1,000-fold with nitric acid to the final concentration of 2% nitric acid, and the samples were analyzed with a ICAP Q quadrupole ICP-MS (Thermo Scientific) at the Department of Earth and Environmental Sciences at Michigan State University.

The general protocol for the AS III assay is described in Table 1.
